# A Dictionary of Terms Employed by the French in Anatomy, Physiology, Pathology, Practical Medicine, Surgery, Midwifery, Pharmacy, Medical Zoology, Botany, and Chemistry; with Their Derivations from the Greek and Latin; Their Synonyms in the Greek, Latin, French, German, and English; and Illustrations in the Different Languages

**Published:** 1836-07-01

**Authors:** 


A Dictionary of Terms employed by the French in Anatomy, Physiology, Pathology, Practical Medicine, Surgery, Midwifery, Pharmacy, Medical Zoology, Botany, and Chemistry; with their Derivations from the Greek and Latin—their Synonyms in the Greek, Latin, French, German, and English; and Illustrations in the different Languages. By *Shirley Palmer*, M.D. Part II. pp. 160, 8vo. Barlow, Birmingham; Longman and Co. 1836.

We return with particular and unfeigned satisfaction to the study of Dr. Palmer's "*Dictionary of Terms;*" and, for the purpose of awakening a congenial spirit in our readers, we open this article with expressing our deliberate and sincere opinion—that this second part of the Doctor's book excels its valuable predecessor in several respects; and, at the same time, it constitutes an additional pledge that, when the work shall have been completed, it will remain a perpetual memorial of its author's learning, industry, high talent, and experience, and likewise prove an inestimable contribution to the advancement and diffusion of useful medical knowledge. Without farther preface, then, we proceed to adduce the evidences on which our opinion is founded; and these evidences shall consist of extracts from the pages of this elaborate and most instructive Dictionary.

Part II. begins with "*Corné,*" and ends with "*Huit de Chiffre,*" the figure-of-8 bandage, and its applications. At "*Corps,*" there is a very comprehensive and important article, containing a brief but luminous view of the different senses in which this term is employed, in natural history, chemistry, anatomy, pathology, and botany. Dr. Palmer describes "*crampe,*" spasm, as a sudden, involuntary, and most painful contraction of one or more muscles, especially those of the lower limbs; and, to his definition, he adds the following practical observations.

"The more ordinary causes of *cramp,* (he says) are exhaustion or fatigue, from inordinate exertion, or an unwonted attitude of the affected muscles. Sometimes, as in *colica pictonum* and *cholera*, the affection is sympathetic of intestinal torpor or irritation. Anodyne frictions, and the application of a tight bandage around the suffering limb, constitute its most effectual remedies. If, as frequently happens, spasm of the stomach, or of any other internal organ or external member, arise from spinal irritation or disease, nothing," the Doctor adds emphatically, and most justly, "but attention to the source of the complaint, can permanently remove or avert the painful consequences." 173.

Dr. Palmer has not exhibited his characteristic precision in his account of "*Craniologie*," skull-talk, "the *doctrine* of the *skull*." Craniology is that section of osteology in which the anatomist describes the entire skull, or its constituent bones and their relations. In the Dictionary, p. 174, however, it is restricted to "an exposition of the inferences which may be drawn from attentive examination of the prominences exhibited by the external surface of the *cranium*, relative to the propensities, moral dispositions, and intellectual faculties of an individual, according to the system of Dr. Gall." Now we infer from his Dictionary, that the learned Doctor's own cranium is composed of stouter stuff, and filled with better brains, than are the skulls of those gentle geniuses—*the Bumpists*—who delight in prattling about prominences; and therefore, as we guess, his definition of "craniology" is a piece of solemn waggery, intended as a quiet reproof for the polemical delinquencies of these facetious little philosophers. According to the system of Dr. Gall, "*craniology*" skull lore, is merely an exposition of the size and shape of the skull, as indicative of the dimensions and configuration of the brain, upon which, as a mould, the cranial bones are consecutively formed.

Cretinism is considered by Dr. P. as a disease endemic in the deeper valleys of different mountainous regions. It is—

"Characterized by extreme moral degradation, and defective configuration (*defective size*) of certain portions of the brain. The disease is hereditary, and may be developed in the children of parents suffering from bronchocele. Residence in the lowest ranges of the Alpine valley, where the air does not circulate freely, is apparently its principal cause. To this may be added, habitual neglect of cleanliness, and a crowded condition and humidity of the dwelling-houses. By some authors it has, without sufficient evidence, been attributed to the dietetic employment of snow-water, and the abuse of alcoholic liquids. That variety of it which originates from malformation is obviously incurable. Prevention consists in properly ventilating, and rendering more dry, the habitations of the poor; in cutting down plantations and draining stagnant waters; and especially in removing children from situations in which the disease is endemic." The Doctor regards "the frequent co-existence of cretinism with bronchocele as a remarkable circumstance in the history of these affections."

On the subject "*crotale*," p. 182, we meet with the following curious observations. The individuals constituting this family (*the crotaloides*) are sufficiently distinguished from all others, as the name imports, by the curious apparatus with which the tail is furnished. It consists of many loosely articulated, horn-like pieces, varying in number according to the age and species, and emitting a distinct and even loud sound on the motion of the animal. One joint of the *rattle* is said to be annually formed, up to a certain period of life. Each superior maxillary bone of this snake exhibits one curved tooth, pointed, perforated in its whole length, and connected with a lobulated gland which secretes the poison. Behind this poison-tooth, there are situated several germs destined to supply its place, in the event of loss or fracture: when at rest, it lies concealed in a fold of the gum. Two muscles, which serve to depress the bone wherein the tooth is immovably fixed, at the moment of bringing the latter into action, exercise a pressure on the secretory organ, and thus propel the poison to the base of the tooth, from which it is conveyed, through the central canal, into the wound inflicted by this formidable instrument. A nictitating membrane exists in the eye: the mouth is large, and the tongue lies partly inclosed in a sheath, and is forked at the extremity. The crotali are viviparous, and exhibit great tenacity of life. The powers of fascination which they in common with some other serpents, are said to exercise on the animals destined to become their prey, have not yet been satisfactorily explained, nor even demonstrated. By some authors, the phenomenon is ascribed to the stupifying effects of the offensive and narcotic effluvium which the bodies of these serpents exhale. They pass rapidly into putrefaction after death; but the tooth, even, retains its virulent properties. The poison is of a green colour. They who recover from its fatal effects, commonly experience, for life, local œdema, periodical pains, hæmorrhages, and weakness or paralysis of the bitten part. The negroes, wild hogs, and birds of prey, feed on the rattle-snake, with impunity.

Crypte, follicle or crypt, is represented as an anatomical term applied to minute, rounded, lenticular, and hollow bodies, situated in the substance of the skin and mucous membranes. They are destined to pour out, upon the surface, from a small orifice, different fluids secreted in the interior. From the *nature* of the *fluid,* they are mucous, sebaceous, unguinous, and ceruminous. From their *situation,* they are cutaneous, ciliary, auricular, labial, palatine, bronchial, œsophageal, gastric, intestinal, vesical, urethral, vaginal and uterine. From their *peculiar disposition,* they are *simple,* as those of the skin and many of the mucous membranes: they are *agglomerate,* as the arytænoid crypts, and those of the palate and lachrymal caruncle: and they are *compound,* as the prostate, amygdalæ, the lacunæ of the rectum and urethra, and the foramen cæcum of the tongue. The crypts serve to keep the various parts in a moist and supple state, and to protect them from the irritating action of the different bodies which come in contact with them. In many diseases, especially those of the mucous membranes, the crypts exhibit great alterations in their figure, volume and secretions. The state of these little organs should never be overlooked, in the processes of a necrotomical inspection.

Dogmatism, at p. 212, is said to be a system or theory, among the ancients, resulting from the application of philosophy, and physical and chemical theories, to medicine. The Dogmatists were, consequently, opposed to the empirics, who professed to take experience alone for their guide. At " *Foie,*" p. 273, the doctor describes the liver as a large abdominal gland, the organ of the biliary secretion; existing under divers modifications of form and structure, in all the animal series, from man to the molluscum. The liver exhibits the peculiarity of receiving, by a distinct apparatus of veins—*the vena porta*—all the returning blood from the chylopoietic organs. The purposes of this disposition are unknown. Venous blood, Dr. P. says, is not essentially requisite for the secretion of bile, since this fluid exists in the mollusca where the vena-portal system is deficient; and it was found in a human subject in whom the vena portarum passed to the vena cava, without entering the liver: besides, the large quantity of blood supplied to the organ by the hepatic artery, would seem to be more than sufficient for the mere purposes of its nutrition.

Dr. Palmer designates homme, homo, man, as the only real biped of the mammiferous class. He alone, gifted with the power of language, is capable of communicating his ideas and emotions, by conventional sounds and signs. His brain is much more complicated, and more fully developed in its anterior portion, than that of other animals. None of the *Quadrumana* possess, like him, a peculiar muscle for the extension of the fore-finger: he alone prepares his food, by subjecting it to the action of fire: and, having acquired the means of protecting his body, by artificial coverings, from the influence of atmospheric vicissitudes, he is fitted to inhabit every accessible region of the globe. The species, the only one of the genus to which he belongs, exhibits the six following races, the Caucasian, Hyperborean, Mongolian, American, Malagan, and Negro or Ethiopian; and each of these is distinguishable by peculiar characters drawn from the figure of the skull, the features of the face, texture of the hair, and colour of the skin.

We might enlarge our list of extracts; but, finding it necessary to close it here, we will conclude with the observation,—that Dr. Palmer's Dictionary should be made a familiar *Vade-Mecum* by every student of the healing art in its manifold departments, and that it will prove an unrivalled acquisition to the libraries of those in whom the pressure of time and care has limited the recollection of elementary medical philology.

